# Therapeutic algorithms for chronic hepatitis C in the DAA era during the current economic crisis: whom to treat? How to treat? When to treat?

**DOI:** 10.1186/1471-2334-12-S2-S3

**Published:** 2012-11-12

**Authors:** Salvatore Petta, Antonio Craxì

**Affiliations:** 1 Sezione di Gastroenterologia, Di.Bi.M.I.S., University of Palermo, Italy

## Abstract

The advent of triple therapy (TT) with first-generation protease inhibitors boceprevir (BOC) and telaprevir (TVR) in addition to pegylated interferon and ribavirin resulted in a significant gain in terms of sustained virological response (SVR) when treating naive or previous treated patients with genotype 1 (G1) chronic hepatitis C (CHC). This gain is partly balanced by the increased complexity of treatment and by the raised costs and risks of therapy, making necessary to optimize the indication to TT.

Specifically, the identification of patient needing to TT over DT, the choice of the more correct therapeutic approach according to baseline and on treatment SVR predictors, and the timing of antiviral treatment, appear key issues to evaluate when considering TVR or BOC-based therapies.

Along this line, further efforts aimed to optimize the current TT regimens are still needed, especially in under-represented groups of patients in phase 3 studies such as those with cirrhosis, where post-marketing data are giving interesting evidences.

## Introduction

In the last few years the treatment picture of patients with genotype 1 (G1) chronic hepatitis C (CHC) is in rapid evolution, due to the discovery of direct acting antiviral (DAA) agents, to be used in combination with PEG-IFN and RBV dual therapy (DT). Specifically, telaprevir (TVR) and boceprevir (BOC), two nonstructural serine (NS3/4) protease inhibitors, are the first DAAs approved for use in the United States and European Union, although many others are in the pipeline [1]. Interestingly the combination of TVR or BOC with PEG-IFN, and RBV (triple therapy - TT) significantly increases the rate of SVR not only in naive [2-4], but also in experienced [5-7] G1 CHC patients. Specifically, when considering previous untreated G1 CHC patients, phase 3 RCTs of TT with BOC (SPRINT-2) [2] or TVR (ADVANCE and ILLUMINATE) [3,4], showed SVR rates ranging from 63% to 75%, therefore highlighting, compared to DT, a gain in SVR rate of about 25%. Similarly, phase 3 trials using TT with BOC (RESPOND-2 and PROVIDE) [5,6] or TVR (REALIZE) (7) and performed on previous treated G1 CHC patients, showed that SVR rates progressively increased from 75% - 86% in relapser (RR), to 52% - 57% in partial responder (PR) and further to 31% - 37% in null responder (NR), resulting in a gain in terms of SVR, compared to DT, of about 55%, 40% and 30%, respectively. Interestingly, it is noteworthy to underline that BOC-based strategies always include an initial 4 weeks lead-in phase with DT followed by TT for a variable treatment duration of both PEG-IFN plus RBV and BOC; by contrast TVR-based strategies always include TT for 12 weeks followed by a variable DT duration, with the possibility to use a lead-in phase only for previous treated patients.

Although these results are very encouraging, the use of these new drugs in clinical practice needs to be carefully evaluated because of such factors as the tolerability profile, the issue of drug-drug interaction, the induction of viral mutations of uncertain significance, and high costs. With these limitations, it appears very relevant not only to identify patients needing TT, but also to use the more correct therapeutic approach and the best timing of treatment.

## Whom to treat?

The use of TT-based strategies in previous untreated or previous treated G1 CHC patients implies a relevant investment in terms of medical and economic resources, also leading during treatment to a significant impairment of the quality of life of the patient. Along this line, to avoid or reduce lack of resources and useless sufferance of the patients, it is very relevant to identify patients who could be advantaged from TT.

When considering a previous untreated G1 CHC patients, firstly it is very relevant to evaluate if the patient is a candidate to antiviral therapy, and more to TT. In this line in fact it is well accepted that antiviral treatment is indicated in all G1 CHC patients with moderate or advanced fibrosis (METAVIR score F2-F3-F4), evaluating individually the indication for treatment of patients with mild fibrosis (METAVIR score F0-F1) according to the likelihood of disease progression [8,9]. However, after evaluating indication to treatment, the next step lies in the attribution to DT versus TT. In this line different literature data showed that naive G1 CHC patients without severe hepatic fibrosis and with IL28B CC genotype have a likelihood of SVR greater than 80% when treated with DT, being similar high rates of SVR also observed in patients achieving a rapid virological response (RVR) (5% of patients with IL28B TT/TC, and 30% of patients with IL28B CC), regardless of the IL28B genotype [10]. Therefore, due to the similar SVR rates observed in IL28BCC naive patients underwent TVR (90%) or BOC-based (80%) TT, it is possible to suggest to avoid TT in these subgroups of patients, due to the fact that the only advantage presented by the TT compared to the DT is a potential shortening of the treatment time in spite of higher costs and higher side effects.

**Figure 1:**
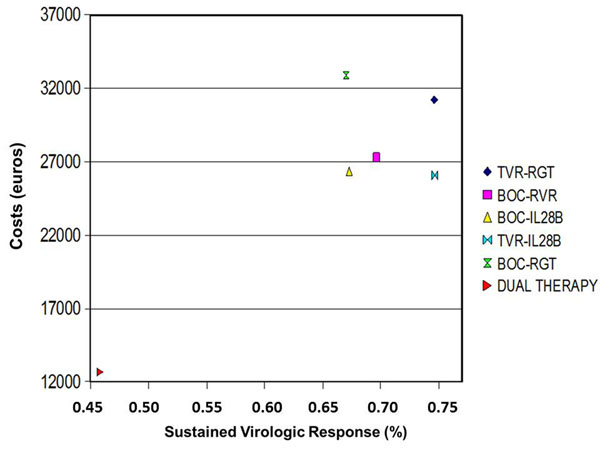
Drug costs (in 2011 euros) and effectiveness evaluated as sustained virologic response. Each symbol represents drug costs versus the proportion of patients who achieve SVR among the competing strategies. (See, Cammà C, et al. Hepatology 2012;56:850-860).

According to all the above, in an era in which resource scarcity will be a prominent issue, we tested, in naïve G1 CHC patients, the cost-efficacy of a combined therapeutic approach (DT and TT) compared to TT in all patients [11]. In line with the above quoted data we observed that, compared with a universal treatment with HCV PI of all untreated G1 CHC patients, using selective treatment strategies guided by RVR for BOC-based therapies, or IL28B genotype for TVR-based strategies, we are able to avoid exposure to HCV PI in 25-33% of patients, reducing costs and risks, and improving benefits [11]. Figure 1 summarized cost-effectiveness of combined and universal strategies compared to DT in naïve G1 CHC patients. Therefore, we recommend using PI-free strategies as first-line therapy in non-cirrhotic patients with IL28B CC genotype or in those who achieve RVR, demanding the use of BOC or TVR-based TT in all the other.

While in naïve G1 CHC patients, IL28B status and RVR achievement are able to identify patients where DT is not inferior to TT in terms of SVR, no DT easy-to-treat sub-groups of previous treated patients were identified. Therefore, in this clinical setting, retreatment of RR or nonresponder to DT is not recommended [8,9], while a rational use of TT is suggested.

## How to treat?

**Figure 2:**
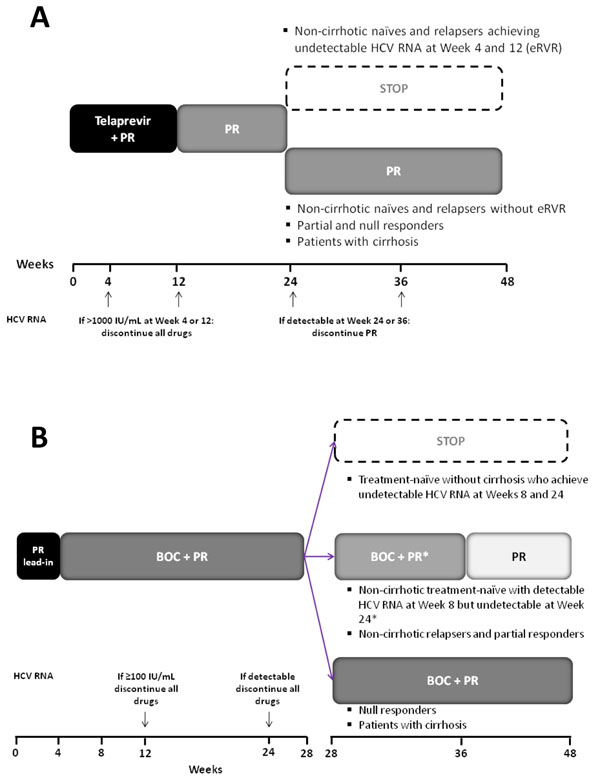
Therapeutic schedules and stopping rules of telaprevir (A) and boceprevir (B) based therapies according to EMA recommendations.

Therapeutic strategies using BOC or TVR are very complex, differ between previous untreated and previous treated patients, differ among these groups according to on treatment response and profile of previous response to DT, and have been further complicated by discrepancies between strategies tested in RCTs and strategies recommended by EMA. Along this line, Figure 2A and 2B show EMA recommendations for treatment of both naïve and experienced G1 CHC patients with BOC and TVR-based strategies, respectively.

In this complex picture, key issues are: a correct assumption of DDAs; the possibility to shorten treatment in a great proportion of patients; correct application of stopping rules; a complete knowledge of factors influencing the achievement of SVR.

A correct taking of DDAs is crucial to maintain a high efficacy of treatment [12,13], minimizing the risk of viral resistance to DAAs. In particular both DAAs, (especially TVR) must be taken with a fat meal of at least 20 g that favours DAAs absorption, and specifically TVR 375 mg tablet must be dosed as 2 pills, 3 times a day, 7–9 hours apart [14], and, similarly, boceprevir 200 mg capsules as 4 pills, 3 times a day, 7–9 hours apart [15].

A very relevant information arising from registrative trials is that treatment can be shortened in a great proportion of patients without compromising the effectiveness of therapy. RCTs on naïve patients [2-4] observed that patients achieving an extended rapid virological response (eRVR), defined as negative HCV-RNA at week 8 maintained through to week 24 for BOC, and as negative HCV-RNA at week 4 maintained through to week 12 for TVR, obtained SVR in more than 90% of cases also reducing treatment duration to 24 and 28 weeks in TVR and BOC-based therapies respectively. These data are of further interest in terms of cost-efficacy and reduction of side-effects related to treatment, if we considered that an eRVR was observed in 44% of patients of SPRINT-2 RCT [2], and in 54% and 63% of ADVANCE [3] and ILLUMINATE [4] studies respectively. Interestingly, in BOC-related therapy, SPRINT-1 study showed the utility of a lead-in phase in increasing the rate of patients achieving an eRVR (62% vs. 38%), finally increasing the number of patients who may benefit from a shortened treatment [16]. Also in previous treated patients there is the possibility to shorten treatment duration. Specifically, only RESPOND-2 study [5] tested this hypothesis showing that patients with an eRVR (46%) receiving 32 weeks of TT after the lead-in phase had an SVR of 86%, similar to the 88% observed in those with eRVR who received additional 12 weeks of DT. However these results, not applicable to patients with severe fibrosis, were not considered by EMA that suggests a 48 weeks therapy also in previous treated patients with eRVR [17]. By contrast, even if a shortened treatment duration was not assessed in REALIZE RCT [7], EMA, following post-hoc analyses of sub-group of patients, suggests to shorten treatment to 24 weeks (12 TT and 12 DT) in RR patients with eRVR [18]. As well as in naïve patients, this recommendation could have relevant management impact considering that eRVR was obtained in 65.5% of RR patients directly treated with TT, and in 87.2% of those treated with a lead-in phase followed by TT. According to all the above a correct evaluation of eRVR is crucial for the optimization of therapy in G1 CHC patients underwent TT. To stress this issue and to give a practice message for clinical practice, a recent post-hoc analysis of RCTs of BOC and TVR investigated the clinical relevance of on treatment detectable but below the assay lower limit of quantitation (detectable/BLOQ) HCV RNA, with respect to undetectable HCV RNA [19]. This study clearly demonstrated that, as performed in RCTs, undetectable HCV RNA must be used to assess the eligibility to a shortened treatment regimen, being detectable/BLOQ associated with a reduction of SVR rates of about 20% compared to patients with undetectable HCV RNA [19].

The early identification of patients without realistic likelihood of SVR to TT is another key issue when treating CHC patients, in order to minimize risk of side-effects, viral resistance and reduce useful costs. Considering TVR, phase 3 studies established as stopping rules HCV RNA levels > 1000 IU/mL at week 4, and a drop by <2 log_10_ at week 12 (stop all drugs) for naïve patients [3,4]; and HCV RNA levels > 100 IU/mL at weeks 4, 6, and 8, or a drop <2 log_10_ at week 12 for previous treated patients [7]. Notwithstanding these data, a re-analysis of EMA, based on few cases of patients with HCV RNA at weeks 4 and 12 between 100 and 1000 IU/mL and achieving SVR, recommended to stopping therapy in both naive and experienced G1 CHC patients treated with TVR if HCV RNA >1000 at week 4 (stop only TVR) or at week 12 (stop all drugs) [18]. Instead AISF Italian guidelines maintained a more conservative approach in experienced patients, suggesting to use the cut-off of 100 IU/mL [20]. Considering BOC, SPRINT-2 [2] and RESPOND-2 [5] studies recommended to stop therapy in case of detectable HCV RNA at week 24 in naive and at week 12 in previous treated G1 CHC patients. As well as with TVR, also for BOC, according to post-hoc analyses of RCTs, EMA modified stopping rules suggesting to stop therapy in case of HCV RNA >100 IU/mL at week 12 in both naive and experienced patients [17]. The rational of this recommendation was also object of a recent scientific paper, aimed to identify uniform stopping rules for all BOC-treated patients [21] for both naive and experienced patients.

The last key issue in the use of TT, is the knowledge of SVR predictors. Considering naïve patients, SPRINT-2 [2] for BOC, and ADVANCE [3] for TVR, showed that the severity of liver fibrosis and IL28B single nucleotide polymorphisms (SNP) affect treatment outcome also in patients underwent TT. In addition, other factors negatively affecting SVR were subtype 1a of viral genotype and black race for both BOC and TVR-based therapies. For a better evaluation of SVR predictors in naïve patients underwent BOC-based TT, a post-hoc analysis has been recently published, including in the model also IL28B SNP, not evaluated by protocol in phase 3 trials. Interestingly, this post-hoc analysis identified in baseline low HCV viral load (≤400,000 IU/mL), IL28B rs 12979860 CC genotype, absence of cirrhosis, HCV genotype 1b, and non black race as independent positive predictors of SVR [22]. In addition, Poordad and colleagues [22], considering data of RCTs on the role of 1log_10_ HCV RNA drop after 4 weeks of DT as predictor of SVR among BOC-treated patients, included in the model of SVR also this variable. Interestingly, the author observed that HCV RNA drop remained significantly associated with SVR, being loosed the effect of IL28B genotype [22]. Considering previous treated G1 CHC patients, data from RESPOND-2 [5] and REALIZE [7] RCTs unequivocally showed that the pattern of the previous response to DT strongly affects the likelihood to achieve SVR after TT, with a progressive increase in SVR rates from NR, to PAR and further to RR. Notwithstanding the pattern of previous response to DT has a great impact on SVR rates in case of retreatment with TT, due to the low availability of these data in all patients in clinical practice, it should be very useful to dispose of on treatment predictors of response to TT. Along this line, data from the lead-in arm of REALIZE [7], and from RESPOND-2 [5], considering together all previously treated G1 CHC patients, showed significantly lower SVR rates in patients with HCV RNA drop <1log_10_ after 4 weeks of DT (33% for TVR, and 34% for BOC), compared to those with a drop >1log_10_ (82% for TVR, and 79% for BOC). In the evaluation of predictors of response to TT among previous treated G1 CHC patients other significant factor needing to be evaluated are the severity of liver fibrosis, and, even if at a les extent, the sub-type of viral genotype, but not IL28B genotype. A further contribution to the evaluation of SVR predictors in previous treated patients, arises from the above quoted post-hoc analysis of Poordad and colleagues [22] on data of RESPOND-2 RCT. In this analysis, after correction for genetic (IL28B), clinical-metabolic, viral and histological variables, the pattern of previous response only, remained independently associated with SVR after BOC-base TT [22]. When adding in the model the 1log_10_ drop HCV RNA at week 4, this last, together with the pattern of response to previous DT, was also an independent predictor [22].

## When to treat?

In the above quoted sections of this review we showed that TT approach adds benefits in terms of SVR in a great proportion of naive G1 CHC patients, and in all previous treated G1 CHC patients. In addition, we also showed that TT was cost-effective compared to DT in both the two groups of patients.

**Figure 3:**
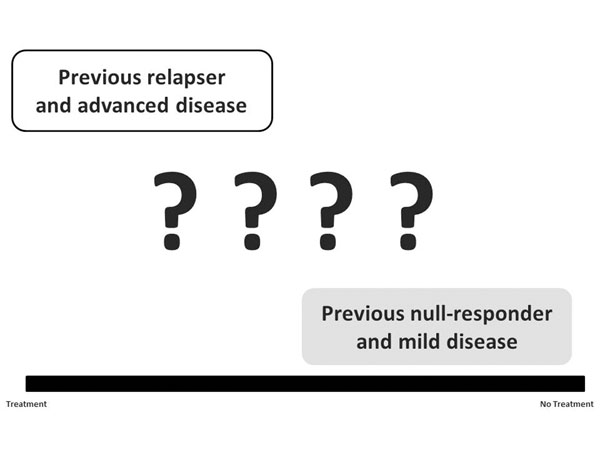
Clinical scenarios where triple therapy or a wait-and-see strategy could be used.

However, it is difficult to translate these data to the conclusion that all G1 CHC patients must be immediately treated with BOC or TVR-based TT, being this issue derived from both economic and clinical considerations. From an economic point of view, in an era in which resource scarcity is and will be a prominent issue, it is not conceivable that all patients are treated with TT, considering the increase in costs due to HCV PI. Along this line, other than to identify patients needing TT with respect to DT (see above), it appears relevant to identify, according to SVR predictors, patients where a treatment is needed in a short time and with a high likelihood of SVR, from patients where the treatment could be deferred and at low likelihood of SVR. In this ongoing debated issue, the 2011 update of the practice guidelines by the American Association of the Study of Liver Disease on Hepatitis C [9] does not recommend any selective allocation of patients to TT with first generation HCV PI. In our opinion, in order to both optimize treatment and reduce risks, two extreme examples could be considered (Figure 3): on one side there are patients with previous RR with advanced CHC/cirrhosis, that are a group of subjects at high risk of liver-related morbidity and mortality in a short time, but also at very high likelihood of SVR if treated with TT; on the other side there are patients with a previous NR and with minimal liver damage, that are a group where liver disease progression is expected after a long time, and at low likelihood of SVR, and therefore where more efficacious drugs are awaited. This can also allow to reduce risk of viral resistances potentially affecting the efficacy of future therapies. Although these two examples are simple, also in these cases some problems exist. In fact, considering the first case, cirrhotic patients are underexpressed in RCTs, and therefore data on efficacy and in particular on safety need to be confirmed. Along this line, ad interim data on expanded access programs [23] of TVR and BOC in patients with advanced HCV liver disease, are showing rates of serious adverse events much higher compared to those reported in RCTs. In addition, in patients with minimal liver damage, the choice to defer antiviral treatment discussed individually with the patients, could be imperfect being the proposed scenarios limited by errors in diagnosing liver damage and predicting progression of fibrosis, by changes in the profile of the patient limiting availability and tolerability of future therapies, and, finally by potential pitfalls of the new drugs in development [24].

In addition, in the middle of these two cases, a great grey area exists, where different factors could affect SVR achievement, and where pros and cons of TT should be carefully evaluated and discussed with the individual patient. Along this line, the availability of individual data from RCTs could allow a more cost-efficacy use of TT.

## Conclusions

RCTs and post-hoc analyses on TVR or BOC-based therapies for naïve or previous treated G1 CHC patients demonstrated a benefit of TT compared to DT. However due the complexity and the high rate of toxicity of these new therapeutic strategies it is crucial to optimize the best candidates to therapy, the best therapeutic schedules, and the more correct timing of treatment. In this line we suggest:

1) To rationally discriminate among naïve G1 CHC patients who could benefit from DT (IL28BCC and/or RVR patients), from those where TT is needed and effective (RR patients), and from those who should await new and more potent drugs (NR patients without significant liver damage).

2) To establish, according to baseline and on treatment SVR predictors, the therapeutic schedule to be applied in the individual patient, to achieve the best results, reducing when possible treatment duration, costs and risks.

## List of abbreviations used

PEG-IFN: Pegylated-interferon; RBV: ribavirin; TVR: telaprevir; BOC: boceprevir; DT: dual therapy; TT: triple therapy.

## Competing interests

The authors declare that they have no competing interests related to the contents of this paper.

## Declarations

Publication of this supplement was partly supported by an unrestricted grant provided by Roche. The articles were independently prepared by the authors with no input from Roche. Roche were not involved in selecting the articles for the supplement. The pegylated interferon (PEG-IFN) treatment mentioned in this article is produced by Roche.
